# Prognostic Value of Cranial Nerve Invasion in T4‐Stage Nasopharyngeal Carcinoma: A Retrospective Cohort Study With a Median Follow‐Up of 136 Months

**DOI:** 10.1002/hsr2.72908

**Published:** 2026-07-25

**Authors:** Cuidai Zhang, Hui Liu, Xuejia Liu, Yihao Ye, Zeman Cai, Zewei Chen, Qizhi Zheng

**Affiliations:** ^1^ Department of Radiation Oncology, Cancer Hospital of Shantou University Medical College Shantou University Shantou China; ^2^ Nasopharyngeal Carcinoma Research Center, Cancer Hospital of Shantou University Medical College Shantou University Shantou China; ^3^ Department of Orthopedics, Cancer Hospital of Shantou University Medical College Shantou University Shantou China; ^4^ Shantou University Medical College Shantou University Shantou China; ^5^ Department of Physical Technology, Cancer Hospital of Shantou University Medical College Shantou University Shantou China

**Keywords:** cranial nerve, locoregional relapse‐free survival, nasopharyngeal carcinoma, overall survival, T4

## Abstract

**Background:**

The objective of this study was to evaluate the prognostic value of cranial nerve invasion (CNI) in T4‐stage nasopharyngeal carcinoma (NPC) patients with nonmetastatic.

**Methods:**

We retrospectively analyzed 299 T4‐stage NPC patients with nonmetastatic disease in the Cancer Hospital of Shantou University Medical College. The primary outcome was the overall survival (OS), and the secondary outcome was locoregional relapse‐free survival (LRRFS).

**Results:**

CNI was observed in 159 (53.2%) NPC patients with T4‐stage disease. The mean follow‐up period was 136 months (range, 3–168 months). Multivariate analyses confirmed that age, pericavernous extension, lower cervical lymph nodes metastasis, CN II and CN X invasion were significant for OS. Multivariate analyses confirmed that prevertebral muscle invasion (PMI) was significant for LRRFS, whereas CNI was not significant for LRRFS. In T4‐stage NPC patients, significant differences were observed between the none and CNI groups in terms of the 5‐ and 10‐year OS rates (70.7%, 61.9% vs. 61.0%, 51.4%; *p* = 0.03), respectively. No significant difference was observed between the none and CNI groups in terms of the 5‐ and 10‐year LRRFS rates (87.4%, 78.1% vs. 88.1%, 79.8%; *p* = 0.71), respectively. In terms of OS, there were significant differences between the none and CN II, CN IX, CN X invasion, multiple CNI, unilateral CNI, and PMI groups, respectively (all *p* < 0.05). Significant differences were observed between the none and CN VII invasion and PMI groups in terms of LRRFS (both *p* < 0.05).

**Conclusions:**

CNI significantly affected T4‐stage NPC patients' survival, although it had no discernible impact on local recurrence. This study elucidates the prognostic significance of CNI among patients with T4‑stage NPC, thereby enabling clinicians to precisely evaluate prognosis and implement optimal therapeutic interventions.

## 1 Introduction

Nasopharyngeal carcinoma (NPC) is a distinct head and neck malignancy with striking geographic prevalence, concentrated in Southeast Asia, Southern China, and North Africa, and accounts for approximately 3% of global head and neck cancer cases [1, 2]. Among NPC stages, T4‐stage disease represents the most locally advanced subset, with inherently poorer prognosis than early T stages [3, 4, 5]. Notably, cranial nerve invasion (CNI) is a core criterion for T4 classification and a critical clinical feature. It occurs in 15%–30% of T4‐stage NPC patients and often presents with disabling symptoms such as facial numbness, diplopia, or dysphagia, which compromise quality of life and may signal aggressive tumor biology [6, 7, 8]. With the widespread adoption of intensity‐modulated radiotherapy (IMRT) and multimodal therapies (e.g., concurrent chemotherapy), the 5‐year overall survival (OS) rate of T4‐stage NPC has improved from < 40% to 50%–60% [9, 10, 11, 12]. However, patients with CNI remain a high‐risk subgroup, as CNI is consistently associated with higher local recurrence rates and shorter OS [7, 13]. Given the clinical significance of OS as a gold‐standard endpoint for evaluating long‐term treatment efficacy, targeted OS analysis for T4‐stage NPC patients with CNI is essential to guide treatment optimization.

Recent studies have advanced understanding of T4‐stage NPC and CNI, particularly in the context of OS. First, treatment efficacy for T4‐stage NPC has been refined. Concurrent chemotherapy significantly improves OS in T4/N2 patients, while induction chemotherapy followed by IMRT may reduce tumor volume and enhance local control [14, 15]. Second, prognostic factor research has identified CNI as an independent predictor of poor OS. Liu et al. [6] demonstrated that T4 patients with MRI‐detected CNI had a 5‐year OS rate of 45.2%, compared to 62.1% for those without CNI, and this finding was validated in a subsequent study [16]. Third, subgroup analyses have begun to explore CNI heterogeneity. Huang et al. [17] reported that mandibular nerve involvement was associated with shorter OS than involvement of other cranial nerves (CNs). Zang et al. [13] noted that incomplete CN palsy recovery after treatment further reduced 5‐year OS by ~20%.

Despite these advances, critical gaps persist in OS analysis for T4‐stage NPC patients with CNI. First, heterogeneity in CNI assessment limits cross‐study comparability. Current studies rely on MRI to define CNI, but criteria are inconsistent, such as nerve thickening versus enhancement [18]. The impact of CNI extent (single vs. multiple CNs) on OS remains understudied [18, 19, 20]. Second, insufficient long‐term OS data for CNI‐positive patients exists. Most studies have follow‐up durations of 3–5 years, but NPC has a risk of late recurrence (> 5 years), making 10‐year OS data critical for evaluating long‐term efficacy [3, 11, 21]. Third, treatment response variability in CNI subgroups is poorly characterized. It is unknown whether patients with multiple CNIs derive greater benefit from intensive chemotherapy (e.g., induction + concurrent chemotherapy) than those with a single CNI, or whether dose escalation improves OS without increasing toxicity [22].

The objective of this study was to systematically investigate the prognostic significance of CNI in patients with T4‐stage NPC, which was a subgroup with inherently poor outcomes, and to identify specific clinical and anatomical factors that could refine risk stratification for OS and LRRFS in the IMRT era. By focusing exclusively on T4‐stage disease, this study aimed to address gaps in prior research, which often included mixed T‐stage cohorts and thus could not isolate the unique prognostic impact of CNI in advanced local disease [6, 16].

## 2 Methods

### 2.1 Patients

Between January 2009 and December 2015, 1396 new NPC patients with nonmetastatic disease were diagnosed in the Cancer Hospital of Shantou University Medical College. All patients were biopsy‐proven, and patients who discontinued treatment were excluded from this study. In this retrospective study, 299 patients with a T4‐stage diagnosis were recruited and restaged using the 8th edition of the AJCC/UICC Cancer Staging System. A comprehensive medical history, physical and neurological examination, hematology and biochemistry profiles, chest radiography, abdominal sonography, whole‐body bone scan, nasopharynx fiberoptic examination, and magnetic resonance imaging (MRI) of the head and neck were all part of the pretreatment evaluation that all the patients underwent.

### 2.2 Diagnostic Criteria for MRI of Infiltration Around CNs

Two experienced radiologists independently reviewed the images. In case of disagreement, they discussed and reached a consensus. The diagnosis could be made if the following signs were present. The determination of these signs must be verified by relevant images from different orientations and different imaging sequences.

The following are examples of direct signs: (i) thickening or enlargement of the CNs or nerve ganglia, or the development of soft tissue nodules or masses; (ii) irregular enhancement of the peripheral CN sheath following enhancement; (iii) enlargement of the cavernous sinus, localized or diffuse thickening of the sinus wall, and irregular enhancement of the local meninges.

The following elements are examples of indirect signs. An MRI reveals bone absorption and destruction; the CN canal is larger than the healthy side, and the normal canal wall bone structure is destroyed with incomplete sclerotic margins. The CN canal's fat gap becomes hazy or vanishes. On the healthy side, the Meckel's cavity is smaller or vanishes, and the trigeminal ganglion is affected by irregular soft tissue nodules.

Cavernous sinus (CS) invasion was defined as tumor infiltration into the CS cavity (involving CN VI and intra‐cavernous internal carotid artery). Invasion of the CS lateral wall (involving CN III/IV) or adjacent Meckel's cave/Gasserian ganglion (CN V) was classified as pericavernous extension, not true CS cavity invasion.

### 2.3 Diagnostic Criteria for Clinical CNI

The diagnostic criteria for clinical CNI are that the patient exhibits symptoms of CN palsy and/or positive clinical CN examination results, which are shown in Table 1.

**Table 1:** Clinical CN examination results and clinical symptoms after CNI of 12 pairs of CNs.

CN no.	Clinical examination results	Clinical symptoms after CNI
I	Unilateral/bilateral inability to identify non‐irritating aromas (e.g., coffee, vanilla) despite intact nasal airflow.	Anosmia (complete loss of smell) or hyposmia (reduced smell); no other focal neurological symptoms.
II	1. Visual acuity: Unilateral decreased acuity (e.g., 20/100) or blindness. 2. Visual fields: Bitemporal hemianopia (if chiasm involved) or monocular field loss. 3. Ophthalmoscopy: Optic disc pallor (atrophy) from nerve compression.	1. Blurred vision, difficulty reading, or complete vision loss in affected eye. 2. Inability to see objects in temporal fields (e.g., “missing cars on the side”). 3. Permanent vision deficit if invasion is long‐standing.
III	1. Pupil: Unilateral dilation (> 4 mm) with absent direct/consensual light reflex. 2. Eye motility: Inability to move eye upward/inward/downward; eye deviates downward/outward. 3. Eyelid: Unilateral ptosis (covers > 50% of iris).	1. Fixed, dilated pupil (warning sign of compressive invasion). 2. Severe diplopia (double vision) with most eye movements; patient avoids eye movement to reduce discomfort. 3. Drooping eyelid obscuring vision; need to manually lift lid.
IV	1. Eye motility: Inability to move eye downward + inward (e.g., can't look at nose). 2. Patient spontaneously tilts head away from the affected side to reduce diplopia.	1. Diplopia worse when reading, descending stairs, or looking down. 2. Compensatory head posture (avoids double vision); neck discomfort from sustained tilt.
V	1. Sensory: Asymmetric loss of touch/pain in V2 (cheeks, upper lip, nasal sidewall) or V3 (chin, lower lip, jaw). 2. Motor (V3): Weak jaw closure; jaw deviates to affected side when opened. 3. Corneal reflex (V1): Absent eye closure if V1 is involved.	1. Focal facial numbness (e.g., “right cheek feels numb”) or trigeminal neuralgia (sharp, shooting pain in V2/V3 distribution). 2. Difficulty chewing; food falls from affected side of mouth. 3. Risk of corneal abrasion (no protective reflex); eye redness/pain.
VI	1. Eye motility: Inability to move eye outward (toward ear); eye remains deviated inward at rest. 2. Patient reports diplopia when looking toward the affected side.	1. Horizontal double vision (e.g., “seeing two cups side‐by‐side” when eating). 2. Avoidance of side gaze (e.g., can't check blind spots while walking); social embarrassment from diplopia.
VII	1. Motor: Unilateral inability to raise eyebrow, close eye tightly (sclera visible), or smile (mouth corner droops). 2. Sensory: Loss of taste on anterior 2/3 of tongue (unilateral).	1. Obvious facial asymmetry; difficulty closing eye (risk of dryness/ulceration). 2. Loss of taste (e.g., “food tastes bland” on one side); reduced quality of life.
VIII	1. Hearing: Unilateral sensorineural hearing loss (whisper test failure; Weber test lateralizes to normal ear). 2. Vestibular: Spontaneous nystagmus; Romberg test: falls/sways with eyes closed.	1. Unilateral hearing loss (e.g., “can't hear phone on left ear”); tinnitus (ringing/buzzing). 2. Vertigo (spinning sensation), nausea; unsteady gait (risk of falls).
IX	1. Gag reflex: Absent contraction of pharyngeal muscles when posterior pharynx is touched (unilateral/bilateral). 2. Sensory: Loss of taste on posterior 1/3 of tongue (unilateral).	1. Dysphagia (difficulty swallowing solids/liquids); increased risk of aspiration (coughing while eating). 2. Loss of taste in posterior tongue; reduced enjoyment of food.
X	1. Pharynx: Uvula deviates to normal side when patient says “ah” (affected side uvula doesn't rise). 2. Voice: Hoarse, breathy voice; aphonia (severe cases). 3. Swallowing: Coughing after drinking water (aspiration).	1. Dysphagia with nasal regurgitation (liquids come out nose); weight loss from reduced intake. 2. Hoarseness (unable to speak loudly); social withdrawal from voice changes. 3. Recurrent aspiration pneumonia (life‐threatening complication).
XI	1. Sternocleidomastoid: Weakness when patient turns head against resistance (e.g., can't push left cheek into hand = right CN XI deficit). 2. Trapezius: Unilateral shoulder droop; inability to shrug affected shoulder.	1. Difficulty turning head (e.g., can't check behind when walking); neck muscle atrophy (late sign). 2. Shoulder pain/stiffness; reduced arm range of motion (e.g., can't lift arm to comb hair).
XII	1. Tongue movement: Tongue deviates to affected side when protruded; weak side‐to‐side movement. 2. Tongue inspection: Unilateral atrophy (wasting) or fasciculations (late).	1. Dysarthria (slurred speech, e.g., “t” sounds like “d”); difficulty communicating. 2. Difficulty eating (tongue can't move food to teeth); weight loss; drooling.

Abbreviations: CN, cranial nerve; CNI, cranial nerve invasion; NPC, nasopharyngeal carcinoma.

### 2.4 Treatment

All patients were treated using IMRT or volumetric modulated arc therapy (VMAT). Patients adopted a radiotherapy scheme with 69.9–70.68 Gy/30–32 fractions, five fractions per week, 2.0–2.35 Gy/fraction. Patients underwent radiotherapy alone or concurrent chemotherapy ± induction chemotherapy ± adjuvant chemotherapy. Chemotherapy regimen was based on cisplatin, with fluorouracil, or docetaxel or paclitaxel.

### 2.5 Follow‐Up

For the first 2 years following the completion of all treatments, patients were seen every 3 months; from the third to the fifth year, they were seen every 6 months; and after that, they were seen once a year. The follow‐up included physical examinations, imaging investigations, nasopharyngoscopies, and hematology testing. OS was computed starting on the day of diagnosis and ending on the date of death or the most recent follow‐up. From the day of diagnosis till the date of local relapse, LRRFS was computed. The outcomes were evaluated on January 31, 2025.

### 2.6 Statistical Analysis

All statistical analyses were performed using IBM SPSS statistical software version 24.0 (IBM, Armonk, NY, USA). Univariate survival analysis was conducted with the Cox proportional hazards regression model and conditional forward stepwise variable selection, and variables with a two‐sided *p*‐value < 0.05 identified in this analysis were further included in the multivariate survival analyses to estimate the effects of potential prognostic factors for OS and LRRFS. The Kaplan–Meier method was utilized in calculating OS and LRRFS. Survival curves were created with the Kaplan–Meier method and compared with the log‐rank test. Statistical significance was set at *p* < 0.05 for all analyses, which were conducted as two‐sided tests.

### 2.7 Ethics and Dissemination

This study was conducted on the basis of the Helsinki Declaration and approved by the Institutional Review Board of the Cancer Hospital of Shantou University Medical College (2025‐II‐092). Informed patient consent was waived because this study was a retrospective study.

## 3 Results

### 3.1 Patient Distribution

The clinical characteristics of the patients are presented in Table 2. Data from 299 patients were collected and analyzed retrospectively in our study. There were 244 (81.6%) men and 55 (18.4%) women, with a mean age of 50.41 ± 12.18 years (range, 15–79 years). The mean follow‐up period was 136 months (range, 3–168 months). Two hundred forty‐seven (82.6%) patients received IMRT and 52 (17.4%) patients received VMAT. Two hundred eighty‐five patients (95.3%) received chemotherapy, and 14 (4.7%) patients received radiotherapy alone. Two hundred eighty‐one (94.0%) patients received concurrent chemotherapy, 30 (10.0%) patients received neoadjuvant chemotherapy, and 25 (8.4%) patients received adjuvant chemotherapy.

**Table 2:** Clinicodemographic characteristics of the patients with T4‐stage NPC (*n* = 299).

Variables	*n* (%)
Age (years, mean [SD])	50.41 (12.18)
Sex	
Male	244 (81.6)
Female	55 (18.4)
Histologic type	
Differentiated non‐keratinizing carcinoma	139 (46.5)
Undifferentiated non‐keratinizing carcinoma	137 (45.8)
Others^a^	23 (7.7)
N stage	
N0	24 (8.0)
N1	74 (24.7)
N2	173 (57.9)
N3	28 (9.4)
Radiation method	
IMRT	247 (82.6)
VMAT	52 (17.4)
Chemotherapy	
No	14 (4.7)
Yes	285 (95.3)
Neoadjuvant chemotherapy	
No	269 (90.0)
Yes	30 (10.0)
Concurrent chemotherapy	
No	18 (6.0)
Yes	281 (94.0)
Adjuvant chemotherapy	
No	274 (91.6)
Yes	25 (8.4)
Skull base invasion	
No	62 (20.7)
Yes	237 (79.3)
Cavernous sinus invasion	
No	234 (78.3)
Yes	65 (21.7)
Pericavernous extension	
No	205 (68.6)
Yes	94 (31.4)
Parotid gland invasion	
No	286 (95.7)
Yes	13 (4.3)
Extensive STI beyond LP invasion	
No	284 (95.0)
Yes	15 (5.0)
PMI	
No	249 (83.3)
Yes	50 (16.7)
Lower cervical lymph nodes metastasis	
No	272 (91.0)
Yes	27 (9.0)
CNI	
No	140 (46.8)
Yes	159 (53.2)
Multiplicity of CNI	
No CNI	140 (46.8)
Single	93 (31.1)
Multiple	66 (22.1)
Sidedness of CNI	
No CNI	140 (46.8)
Unilateral	156 (52.2)
Bilateral	3 (1.0)
CN I	
No	293 (98.0)
Yes	6 (2.0)
CN II	
No	272 (91.0)
Yes	27 (9.0)
CN III	
No	270 (90.3)
Yes	29 (9.7)
CN IV	
No	291 (97.3)
Yes	8 (2.7)
CN V	
No	216 (72.2)
Yes	83 (27.8)
CN V1	
No	287 (96.0)
Yes	12 (4.0)
CN V2	
No	220 (73.6)
Yes	79 (26.4)
CN V3	
No	281 (94.0)
Yes	18 (6.0)
CN VI	
No	234 (78.3)
Yes	65 (21.7)
CN VII	
No	294 (98.3)
Yes	5 (1.7)
CN VIII	
No	297 (99.3)
Yes	2 (0.7)
CN IX	
No	283 (94.6)
Yes	16 (5.4)
CN X	
No	292 (97.7)
Yes	7 (2.3)
CN XII	
No	269 (90.0)
Yes	30 (10.0)

Abbreviations: CN, cranial nerve; CNI, cranial nerve invasion; IMRT, intensity‐modulated radiotherapy; LP, the lateral surface of the lateral pterygoid muscle; NPC, nasopharyngeal carcinoma; PMI, prevertebral muscle invasion; SD, standard deviation; STI, soft tissue infiltration; VMAT, volumetric modulated arc therapy.

^a^ Others include squamous cell carcinoma, low differentiated squamous cell carcinoma and moderately differentiated squamous cell carcinoma.

Of 299 NPC patients with T4‐stage, 159 (53.2%) patients initially presented with CNI. Ninety‐three (31.1%) patients had single CNI and 66 (22.1%) patients had multiple CNIs. A total of 156 (52.2%) patients had unilateral CNI and 3 (1.0%) patients had bilateral CNI. Six (2.0%) patients had CN I invasion and 27 (9.0%) patients had CN II invasion. Twenty‐nine (9.7%) patients had CN III invasion and 8 (2.7%) patients had CN IV invasion. Eighty‐three (27.8%) patients had CN V invasion, 12 (4.0%) had CN V1 invasion, 79 (26.4%) had CN V2 invasion, and 18 (6.0%) had CN V3 invasion. Five (1.7%) patients had CN VII invasion, and sixty‐five (21.7%) patients had CN VI invasion. CN VIII invasion was found in two individuals (0.7%) and CN IX invasion in 16 patients (5.4%). Thirty (10.0%) patients had CN XII invasion, while seven (2.3%) patients had CN X invasion.

A total of 299 patients were analyzed. Anosmia was present in 0.3% (*n* = 1) of patients. Blurred vision, absent direct or consensual light reflex, ptosis of eyelid, and diplopia were observed in 4.0% (*n* = 12), 0.7% (*n* = 2), 3.3% (*n* = 10), and 21.7% (*n* = 65) of patients, respectively. Focal facial numbness was found in 27.8% (*n* = 83) of patients. Mouth corner droops occurred in 1.3% (*n* = 4). Absent gag reflex, dysphagia, and tongue muscle atrophy were each present in 0.3% (*n* = 2). Coughing after drinking water was reported in 0.3% (*n* = 1). Tongue deviation on protrusion and dysarthria were detected in 5.0% (*n* = 15) and 2.3% (*n* = 7) of patients, respectively (Table S1).

### 3.2 Univariate Cox Regression Analysis

Univariate analysis confirmed that chemotherapy, radiotherapy technology, age, skull base invasion, pericavernous extension, parotid gland invasion, extensive soft tissue infiltration beyond the lateral surface of the lateral pterygoid muscle invasion, prevertebral muscle invasion (PMI), lower cervical lymph nodes metastasis, multiplicity of CNI, sidedness of CNI, CN II, IX, and X invasion were significant for OS (Table 3). Univariate analysis demonstrated that PMI and CN VII invasion were significant for LRRFS (Table 3).

**Table 3:** Summary of the univariable Cox regression analysis of prognostic factors for OS and LRRFS.

Variables	OS		LRRFS	
	*p* value	HR (95% CI)	*p* value	HR (95% CI)
Age	0.008	1.02 (1.0–1.04)	0.39	0.99 (0.96–1.01)
Sex	0.29	0.76 (0.45–1.27)	0.31	1.45 (0.71–2.98)
N stage	0.54	1.08 (0.84–1.40)	0.31	1.26 (0.81–1.96)
Histologic type	0.71	0.94 (0.69–1.28)	0.26	1.33 (0.81–2.20)
Radiation method	0.01	0.45 (0.24–0.84)	0.83	1.09 (0.50–2.37)
Chemotherapy	0.011	0.41 (0.21–0.81)	0.43	21.21 (0.01–44,835.08)
Skull base invasion	0.004	2.33 (1.31–4.15)	0.23	1.70 (0.71–4.08)
CS invasion	0.50	1.16 (0.75–1.79)	0.54	0.77 (0.34–1.75)
Pericavernous extension	0.03	1.51 (1.03–2.20)	0.09	1.73 (0.91–3.28)
Intracranial invasion	0.40	0.82 (0.52–1.29)	0.75	0.88 (0.42–1.86)
Orbit invasion	0.35	1.28 (0.76–2.15)	0.94	1.04 (0.41–2.66)
Parotid gland invasion	0.003	2.86 (1.44–5.66)	0.39	1.87 (0.45–7.77)
Extensive STI beyond LP invasion	0.03	2.11 (1.07–4.18)	0.62	1.43 (0.34–5.97)
PMI	0.002	1.96 (1.28–3.01)	0.001	3.21 (1.64–6.27)
Lower cervical lymph nodes metastasis	0.02	1.84 (1.08–3.13)	0.65	1.28 (0.45–3.59)
Multiplicity of CNI	0.018	1.32 (1.05–1.65)	0.83	1.05 (0.70–1.55
Sidedness of CNI	0.023	1.52 (1.06–2.17)	0.81	1.08 (0.59–1.97)
CN I	0.36	1.72 (0.55–5.42)	0.55	1.83 (0.25–13.36)
CN II	0.03	1.82 (1.056–3.13)	0.50	0.61 (0.15–2.55)
CN III	0.86	1.06 (0.57–1.97)	0.64	0.75 (0.23–2.44)
CN IV	0.09	2.18 (0.89–5.34)	0.97	1.04 (0.14–7.57)
CN V	0.20	1.30 (0.87–1.93)	0.16	1.60 (0.83–3.08)
CN VI	0.50	1.16 (0.75–1.79)	0.54	0.77 (0.34–1.75)
CN VII	0.13	2.43 (0.77–7.66)	0.01	6.17 (1.48–25.74)
CN VIII	0.50	1.97 (0.28–14.10)	0.78	0.05 (0.000–55,271,941.90)
CN IX	0.03	2.16 (1.09–4.27)	0.20	2.18 (0.67–7.07)
CN X	< 0.001	5.12 (2.24–11.70)	0.68	0.05 (0.000–87,127.57)
CN XII	0.76	1.10 (0.60–2.00)	0.07	2.12 (0.94–4.82)

Abbreviations: CN, cranial nerve; CNI, cranial nerve invasion; CS, cavernous sinus; HR, hazard ratio; LP, the lateral surface of the lateral pterygoid muscle; LRRFS, locoregional relapse‐free survival; OS, overall survival; PMI, prevertebral muscle invasion; STI, soft tissue infiltration.

### 3.3 Multivariate Cox Regression Analyses

Multivariate analyses confirmed that age, pericavernous extension, lower cervical lymph nodes metastasis, CN II and CN X invasion were significant for OS (Table 4). Multivariate analyses confirmed that PMI was significant for LRRFS, while CNI was not significant for LRRFS (Table 4).

**Table 4:** Summary of the multivariate Cox regression analyses of prognostic factors for OS and LRRFS.

Variables	OS		LRRFS	
	*p* value	HR (95% CI)	*p* value	HR (95% CI)
Age	0.01	1.02 (1.01–1.04)		
Radiation method	0.10	0.57 (0.30–1.10)		
Chemotherapy	0.16	0.59 (0.28–1.23)		
Skull base invasion	0.39	1.31 (0.71–2.43)		
Pericavernous extension	0.05	1.55(1.01–2.40)		
Parotid gland invasion	0.21	2.14 (0.65–7.09)		
Extensive STI beyond LP invasion	0.69	1.27 (0.40–4.06)		
PMI	0.21	1.36 (0.84–2.19)	0.003	2.90 (1.44–5.87)
Lower cervical lymph nodes metastasis	0.05	1.74 (1.00–3.03)		
Multiplicity of CNI	0.24	0.70 (0.39–1.26)		
Sidedness of CNI	0.09	2.01 (0.89–4.53)		
CN II	0.01	2.32 (1.19–4.51)		
CN VII			0.17	2.87 (0.64–12.84)
CN IX	0.06	2.20 (0.98–4.93)		
CN X	0.02	3.62 (1.27–10.32)		

Abbreviations: CN, cranial nerve; CNI, cranial nerve invasion; HR, hazard ratio; LP, the lateral surface of the lateral pterygoid muscle; LRRFS, locoregional relapse‐free survival; OS, overall survival; PMI, prevertebral muscle invasion; STI, soft tissue infiltration.

### 3.4 Survival Analysis

**Figure 1:**
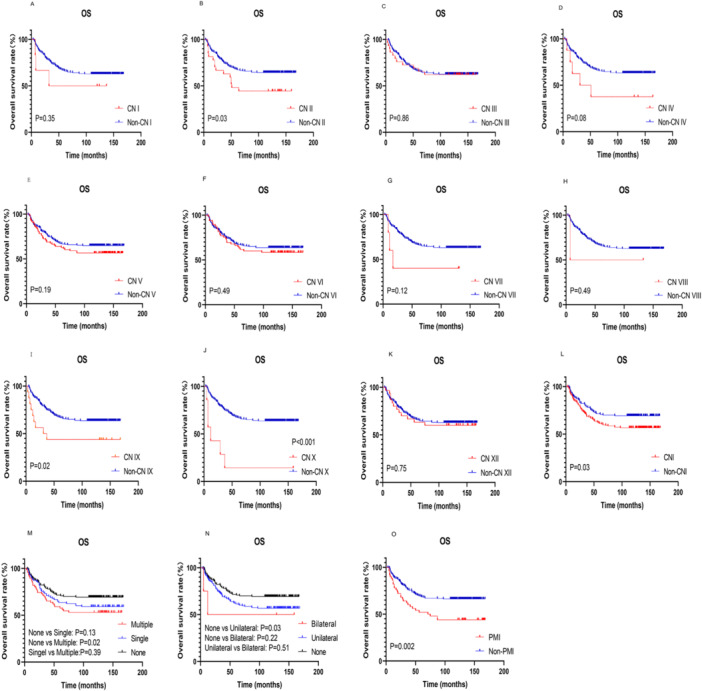
Prognostic stratification of OS among T4‐stage patients with various CN classifications. (A, C–H, K) No significant difference was observed between the none and CN I, III, IV, V, VI, VII, VIII, XII invasion groups. (B, I, J) Significant differences were observed between the none and CN II, IX, X invasion groups. (L, O) Significant differences were observed between the none and CNI, PMI groups. (M) Significant differences were observed between the none and multiple CNI groups. (N) Significant differences were observed between the none and unilateral CNI groups. CN, cranial nerve; CNI, cranial nerve invasion; OS, overall survival; PMI, prevertebral muscle invasion.

**Figure 2:**
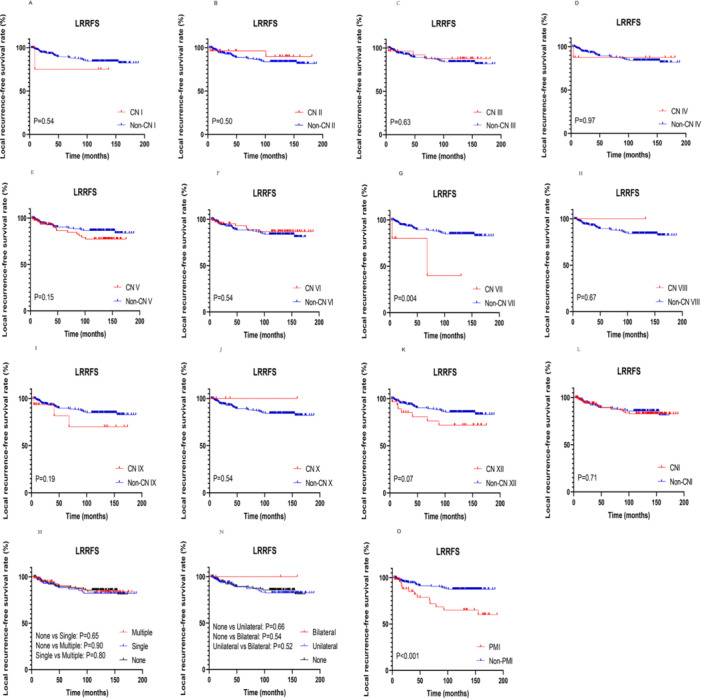
Prognostic stratification of LRRFS among T4‐stage patients with various CN classifications. (A–F, H–K) Significant difference was not observed between the none and CN I, II, III, IV, V, VIII, IX, X, XII invasion groups. (L) Significant difference was not observed between the none and CNI groups. (M) Significant difference was not observed between the none and multiplicity of CN groups. (N) Significant difference was not observed between the none and sidedness of CNI groups. (G) Significant differences were observed between the none and CN VII invasion groups. (O) Significant differences were observed between the none and PMI groups. CN, cranial nerve; CNI, cranial nerve invasion; LRRFS, locoregional relapse‐free survival; PMI, prevertebral muscle invasion.

The 5‐ and 10‐year OS rates for T4‐stage NPC patients presented significant differences between the none and CNI groups (70.7%, 61.9% vs. 61.0%, 51.4%; *p* = 0.03, Figure 1L), respectively. The 5‐ and 10‐year LRRFS rates (87.4%, 78.1% vs. 88.1%, 79.8%; *p* = 0.71, Figure 2L) did not significantly differ between the none and CNI groups. In terms of OS, there were significant differences between the none and CN II, CN IX, CN X invasion, multiple CNI, unilateral CNI, and PMI groups, respectively (all *p* < 0.05). Significant differences were observed between the none and CN VII invasion and PMI groups in terms of LRRFS (both *p* < 0.05). Prognostic stratification of OS and LRRFS among T4‐stage patients with various CN classifications was depicted in Figures 1 and 2, respectively.

### 3.5 Discussion

Among 299 retrospective T4‐stage NPC patients, 53.2% (*n* = 159) exhibited CNI. This invasion was significantly associated with worse long‐term survival but not with LRRFS. Multivariate analyses further identified age, pericavernous extension, lower cervical lymph node metastasis, and involvement of CN II/X as independent predictors of OS, while PMI emerged as the sole independent prognostic factor for LRRFS. These findings align with foundational studies [6, 16] that established CNI as an adverse prognostic marker for OS in NPC but diverge from earlier work [23] that suggested a link between CNI and reduced LRRFS. The discrepancy in LRRFS outcomes is likely attributable to the widespread adoption of IMRT (82.6%) or VMAT (17.4%) with concurrent chemotherapy (94.0%) in our study. Unlike 3D conformal radiotherapy, IMRT enables precise dose delivery to skull base and CN tracts, minimizing locoregional failure even in patients with CNI [7, 24, 25]. There is a trend supported by recent IMRT‐focused studies [11, 12]. Additionally, the extended follow‐up period (mean 136 months) in our study provides more robust long‐term survival data compared to shorter follow‐up (≤ 5 years) in many prior reports, enhancing the reliability of conclusions regarding OS and LRRFS [6, 21].

In line with Liu et al. [6], who reported that CNI independently predicts poor OS in NPC, our multivariate analyses confirmed CN‐related factors (e.g., involvement of CN II/X) as OS predictors, reinforcing the clinical relevance of CNI subtyping. Similarly, Kong et al. [11] noted that concurrent chemotherapy improves outcomes in T4‐stage NPC, which aligns with our finding that 95.3% of patients receiving chemotherapy (mostly concurrent) had survival patterns consistent with enhanced disease control. However, our study advances beyond these works by quantifying the prognostic impact of specific CN subtypes. For instance, CN V involvement (27.8% of cases, with CN V2 being the most common branch, 26.4%) was not an independent OS predictor, whereas CN II (9.0%) and CN X (2.3%) involvement were independent OS predictors. This observation is rarely addressed in prior studies [19, 26]. Cui et al. [19] focused on trigeminal nerve palsy but did not explore its prognostic weight relative to other nerves, highlighting our study's unique emphasis on subtype‐specific risk stratification.

Regarding OS, our results align with landmark studies that established CNI as a poor prognostic factor [6, 11, 12, 16]. Liu et al. [6] first reported via MRI‐detected CNI that NPC patients with CNI had a 2.1‐fold higher risk of death. This finding was validated by Liu et al. [16] in a larger cohort (*n* = 864), where CNI remained an independent predictor of OS (HR = 1.32, *p* = 0.002). Extending these observations to T4‐specific disease, Huang et al. [7] noted that CNI was associated with a 30% reduction in 5‐year OS in T4 patients treated with conventional radiotherapy. Takiar et al. [12] later confirmed this trend in the IMRT era (5‐year OS: 58% vs. 72% for T4‐stage patients with CNI vs. without CNI). Our data presented a 9.7% and 10.5% absolute reduction in 5‐ and 10‐year OS, respectively, which reinforces this cross‐study consistency. The inclusion of 10‐year follow‐up, which is rare in CNI research [11, 27], confirms that CNI's adverse impact is not transient but persists long‐term.

Notably, this study advances prior work by delineating subtype‐specific CNI prognosticators, a gap addressed in only a handful of recent studies. Zhu et al. [28] proposed that skull base foramen invasion subclassification improves T‐category refinement, but their analysis focused on anatomical sites (e.g., foramen ovale) rather than individual CNs. In contrast, our multivariate model identified CN II and CN X invasion as independent OS predictors. Our study complements the study of Li et al. [29], who reported that mandibular nerve (CN V3) involvement correlated with worse outcomes (HR = 1.56, *p* = 0.003) but did not evaluate other CNs. The prognostic significance of CN II invasion may reflect its anatomical proximity to the cavernous sinus, where tumor spread is more likely to involve critical intracranial structures and increase systemic relapse risk [8, 19, 30]. Similarly, CN X invasion may indicate more extensive submucosal spread, a known marker of aggressive disease [31, 32].

In terms of LRRFS, our finding that CNI was not associated with locoregional failure contrasts with early studies but aligns with contemporary IMRT research. Li et al. [23] initially suggested that CNI increased LRRFS risk (5‐year LRRFS: 52% vs. 78%, *p* < 0.001) in a cohort treated with conventional radiotherapy, but this was attributed to suboptimal target volume coverage of CN pathways [24, 33]. In the IMRT era, Kong et al. [15] demonstrated that improved dose conformity to skull base tracts reduced 5‐year LRRFS failure rates in T4 NPC to < 15%, regardless of CNI status, and our data (5‐year LRRFS: 87.4% for both groups) confirm this trend. Our multivariate analyses further identified PMI as the sole independent LRRFS predictor, which was consistent with Liang et al. [34], who reported that prevertebral space involvement correlated with a 2.3‐fold higher locoregional relapse risk (*p* = 0.004) due to the challenge of delivering ablative doses to this area without damaging adjacent spinal cord or vasculature [25, 35]. This distinction (CNI for OS vs. PMI for LRRFS) clarifies the differential roles of anatomical invasion sites, a nuance not previously emphasized in CNI‐focused studies [6, 16]. Takiar et al. [12] reported that T4‐stage NPC treated with IMRT achieves favorable local control, but they did not stratify by CNI status. Our study fills this gap by demonstrating that even with modern radiotherapy, CNI does not compromise LRRFS. It is likely due to IMRT/VMAT's ability to deliver precise doses to skull base regions and reduce normal tissue toxicity [12, 25]. This contrasts with Fei et al. [22], who suggested that residual lesions after IMRT require boost doses to improve local control, but our data show no residual LRRFS deficit in CNI patients, possibly because concurrent chemotherapy (94.0% of our cohort) reduced micrometastatic disease that might otherwise lead to recurrence.

Most previous CNI prognostic studies included mixed T‐stage populations, which may dilute the prognostic signal of CNI in advanced disease, while our exclusive focus on T4 patients eliminates stage‐related confounding [6, 12, 16]. Additionally, the identification of CN II/X as independent OS predictors provides novel, actionable insights for risk stratification. It is an advance over studies that treated CNI as a binary variable and did not explore subtype‐specific effects [6, 7].

### 3.6 Limitations

The small sample size in the VMAT group is one of the study's limitations because our medical center only began employing VMAT as a treatment in late 2012. To assess the long‐term results and problems, a bigger patient group and a longer follow‐up period are required. Chemotherapy was not consistent in our investigations because they were retrospective in nature. Furthermore, CNIs might occasionally have mild symptoms or no symptoms at all. There are certain limitations to evaluating CNIs using clinical symptoms and physical testing. Consequently, a more precise and thorough neurological examination is needed.

## 4 Conclusion

According to this study, CNI significantly affected T4‐stage NPC patients' survival, although it had no discernible impact on T4‐stage patients' local recurrence. Age, pericavernous extension, lower cervical lymph nodes metastasis, CN II and CN X invasion were the independent prognostic factors for OS. Prevertebral muscle invasion was the independent prognostic factor for LRRFS. It is important to prioritize aggressive systemic therapy for T4 patients with CNI and optimize radiotherapy dose to prevertebral muscles to improve LRRFS.

This study clarifies the long‐standing clinical ambiguity about the prognostic value of CNI and its related characteristics in T4‐stage NPC, filling the research gap, enriching advanced NPC prognosis evidence, and helping clinicians accurately evaluate patients' disease progression risk to take appropriate treatment measures.

## Author Contributions

**Cuidai Zhang:** conceptualization, methodology, software, data curation, investigation, writing – original draft, writing – review and editing. **Hui Liu:** data curation, investigation, formal analysis, software. **Xuejia Liu:** data curation, formal analysis. **Yihao Ye:** data curation, formal analysis. **Zeman Cai:** data curation, formal analysis. **Zewei Chen:** conceptualization, investigation, project administration, formal analysis, supervision. **Qizhi Zheng:** conceptualization, methodology, project administration, investigation, validation.

## Funding

The authors have nothing to report.

## Conflicts of Interest

The authors declare no conflicts of interest.

## Transparency Statement

The lead author, Cuidai Zhang, affirms that this manuscript is an honest, accurate, and transparent account of the study being reported; that no important aspects of the study have been omitted; and that any discrepancies from the study as planned (and, if relevant, registered) have been explained.

## Supporting information


Supporting File

## Data Availability

The data that support the findings of this study are available from the corresponding author upon reasonable request. All authors have read and approved the final version of the manuscript. Qizhi Zheng and Zewei Chen had full access to all of the data in this study and take complete responsibility for the integrity of the data and the accuracy of the data analysis.
