# Supplementary material for: Prognostic Value of Cranial Nerve Invasion in T4‐Stage Nasopharyngeal Carcinoma: A Retrospective Cohort Study With a Median Follow‐Up of 136 Months
Source: Health Sci Rep. 2026 Jul 25;9(8):e72908. doi: 10.1002/hsr2.72908 (PMC13401712; doi:10.1002/hsr2.72908)
Supplement: Supplementary file 1 — Supporting File [file HSR2-9-e72908-s001.docx]

Supplement table 1. Clinical examination results and clinical symptoms of the T4 stage NPC patients (n = 299).

| Variables |  | n (%) |
| --- | --- | --- |
| Anosmia | No | 298 (99.7) |
|  | Yes | 1 (0.3) |
| Blurred vision | No | 287 (96.0) |
|  | Yes | 12 (4.0) |
| Absent direct/consensual light reflex | No | 297 (99.3) |
|  | Yes | 2 (0.7) |
| Ptosis of eyelid | No | 289 (96.7) |
|  | Yes | 10 (3.3) |
| Diplopia | No | 234 (78.3) |
|  | Yes | 65 (21.7) |
| Focal facial numbness | No | 216 (72.2) |
|  | Yes | 83 (27.8) |
| Mouth corner droops | No | 295 (98.7) |
|  | Yes | 4 (1.3) |
| Absent gag reflex | No | 297 (99.3) |
|  | Yes | 2 (0.3) |
| Dysphagia | No | 297 (99.3) |
|  | Yes | 2 (0.3) |
| Coughing after drinking water | No | 298 (99.7) |
|  | Yes | 1 (0.3) |
| Tongue deviates to affected side when protruded | No | 284 (95.0) |
|  | Yes | 15 (5.0) |
| Tongue muscle atrophy | No | 297 (99.3) |
|  | Yes | 2 (0.3) |
| Dysarthria | No | 292 (97.7) |
|  | Yes | 7 (2.3) |

Abbreviations: NPC = nasopharyngeal carcinoma.
